# Regulation of the Innate Immune Response by Fibronectin: Synergism between the III-1 and EDA Domains

**DOI:** 10.1371/journal.pone.0102974

**Published:** 2014-07-22

**Authors:** Rhiannon Kelsh, Ran You, Carol Horzempa, Mingzhe Zheng, Paula J. McKeown-Longo

**Affiliations:** Center for Cell Biology & Cancer Research, Albany Medical College, Albany, New York, United States of America; Medical University of South Carolina, United States of America

## Abstract

Fibronectin is a critical component of the extracellular matrix and alterations to its structure will influence cellular behavior. Matrix fibronectin is subjected to both mechanical and biochemical regulation. The Type III domains of fibronectin can be unfolded in response to increased cellular contractility, included or excluded from the molecule by alternative splicing mechanisms, or released from the matrix by proteolysis. Using Inflammatory Cytokine microarrays we found that the alternatively spliced fibronectin Type III domain, FnEDA, and the partially unfolded III-1 domain, FnIII-1c, induced the expression of a multitude of pro-inflammatory cytokines in human dermal fibroblasts, most notably CXCL1-3, IL-8 and TNF-α. FnIII-1c, a peptide representing an unfolded intermediate structure of the first Type III domain has been shown to initiate the toll-like receptor-4 (TLR4)-NFκB-dependent release of cytokines from human dermal fibroblasts (You, et al., J. Biol. Chem., 2010). Here we demonstrate that FnIII-1c and the alternatively spliced FnEDA domain induce a TLR4 dependent activation of p38 MAP kinase and its downstream effector, MAPKAP Kinase-2 (MK-2), to regulate cytokine expression in fibroblasts. RT-qPCR analysis indicated that the p38-MK-2 pathway regulates IL-8 mRNA stability. Interestingly, addition of FnIII-1c and FnEDA synergistically enhanced TLR4-dependent IL-8 release. These data indicate that Fn contains two Type III domains which can activate TLR signaling to induce an inflammatory response in fibroblasts. Furthermore, our data identifies the NF-κB and p38/MK2 signaling pathways as transducers of signals initiated in response to structural changes in fibronectin.

## Introduction

The extracellular matrix (ECM)^2^ plays an essential role in tissue organization and function. The interaction of cells with the ECM depends primarily on integrin receptors which convey both structural and chemical information into the cell, while reciprocally remodeling the matrix through their regulation of ECM assembly and turnover. How the cell integrates the mechanical and biochemical information present in the ECM to impact cellular function is not well understood. The fibronectin matrix is a complex network of polymerized fibers which undergoes extensive remodeling during the processes of development and tissue repair and during the progression of most diseases (reviewed in [1]). Fibronectin is a plasma protein synthesized by the liver which undergoes a cell-dependent polymerization into a fibrillar extracellular matrix in most tissues. Both plasma-derived and local synthesis by resident stromal cells contribute to the fibronectin which makes up the tissue matrix. The secondary structure of the fibronectin molecule is organized into individually folded domains, termed Types I, II and III, which represent regions of amino acid homology. The Type I and II domains are stabilized by the presence of intra-domain disulfide bonds, while the Type III domains are structurally more labile and subject to mechanical unfolding [2]. There are 15–17 Type III domains in fibronectin and the biological role of many of these domains is not well understood.

Both normal and pathological remodeling of the fibronectin matrix occurs at the biochemical and mechanical level. Typically stromal cells such as fibroblasts are the major orchestrators of fibronectin remodeling. At the biochemical level, fibronectin synthesized by fibroblasts undergoes alternative splicing resulting in the increased expression of two Type III domains termed EDA and EDB [3]. These fibronectin isoforms are seen only when tissues are being actively remodeled, such as during periods of tissue repair and in association with fibrosis and inflammation. The fibronectin matrix is sensitive to a variety of proteases which release bioactive fragments from the matrix thereby regulating cell adhesion [4], apoptosis [5], and the release of inflammatory mediators and proteases [6]. In response to mechanical forces, the Type III domains of fibronectin can unfold to either reveal or inhibit biologically active sites within the matrix [7]. Mechanically-regulated sites within the matrix have been implicated in fibronectin polymerization [8]–[10], motogenic activity [11], cell adhesion [12]–[15], growth factor binding [16], and bacterial colonization [17].

Extracellular matrix molecules, including fibronectin, have been identified as activators of toll-like receptors (TLRs). TLRs are a family of transmembrane receptors which function as regulators of the innate immune system and mediate the release of inflammatory cytokines in response to pathogens and damaged tissue [18]. TLRs, initially identified on myeloid cells as pattern recognition receptors which recognized bacterial pathogens or PAMPS (Pathogen-associated molecular patterns), have now been found on most tissue cells including skin fibroblasts [19]. TLRs can also become activated in response to intrinsic molecules in the absence of pathogens. Intracellular molecules released from damaged tissue as well as fragments of extracellular matrix molecules have been termed DAMPs (Damage-associated molecular patterns). These molecules can also elicit an immune response following tissue injury or in response to the changes in tissue composition and organization which accompany a variety of pathologies. Two domains in fibronectin have been reported to activate TLR signaling. The extra Type III domain, FnEDA, stimulates TLR4 dependent cytokine release from mast cells and T cells [20], [21]. We have previously shown that a partially unfolded intermediate structure of the first Type III (III-1) domain of fibronectin (FnIII-1c) activates TLR4 and TLR2 mediated cytokine release from skin and lung fibroblasts, respectively [22], [23]. This intermediate structure of the unfolded III-1 domain has been predicted to occur in response to cellular generated contractile force and by metalloprotease cleavage. The NMR structure of the III-1 domain revealed a β sandwich containing 7 strands organized into two β sheets (strands G, F, C, D and A, B, E). Simulations of III-1 unfolding by steered molecular dynamics identified a prominent unfolding intermediate in which the A and B strands are separated from the folded core, ^C-G^FnIII-1 [24]. Based on their similar NMR structures, ^C-G^FnIII-1, the structure predicted to form in response to mechanical force can be recapitulated in a recombinant peptide, FnIII-1c, previously identified and characterized by the Ruoslahti laboratory [25]. The FnIII-1c peptide may also be generated by MMP2 cleavage which gives rise to a peptide with the same N-terminus and similar molecular mass as seen in FnIII-1c [26].

In dermal fibroblasts, FnIII-1c induced the expression of IL-8 and TNF-α which was dependent on NF-κB and accompanied by the activation of p38 MAP kinase [23], [27], [28]. The purpose of the present study was to define the relationship of p38 activation to the TLR4-dependent induction of IL-8 by FnIII-1c and to determine whether the EDA domain of fibronectin could also induce cytokine expression in dermal fibroblasts. We show that the NFκB dependent induction of IL-8 in dermal fibroblasts by FnIII-1c is modulated by a p38/MK2 signaling axis which controls IL-8 message stability. We further show that FnEDA and FnIII-1c each activate the same signaling pathways in dermal fibroblasts to induce a similar signature of inflammatory genes. In addition, the effects of FnEDA and FnIII-1c on IL-8 expression are synergistic suggesting that the simultaneous changes in cell contractility, alternative splicing and pericellular proteolysis can lead to an enhanced inflammatory response by resident fibroblasts within a tissue and implicate the topography of the fibronectin matrix as having an important role in the regulation of the innate immune system during periods of tissue remodeling.

## Materials and Methods

### Reagents and Antibodies

Reagents were purchased from Sigma Chemical Co. (St. Louis, MO) unless otherwise indicated. MK-2 Inhibitor III, BAY 11-7082 and human Tenascin-C were purchased from EMD Millipore (Billerica, MA). PS-1145 (NF-κB inhibitor) was purchased from Sigma. Blocking antibodies to human TLR4 were purchased from R&D Systems (Minneapolis, MN). Rabbit polyclonal antibodies to phospho-p38 MAP Kinase (Thr180/Tyr182), total p38 MAPK, phospho-HSP27 (Ser82), total MAPKAPK-2 as well as the rabbit monoclonal antibody to phospho-MAPKAPK-2 (Thr334) were purchased from Cell Signaling Technology (Danvers, MA). Antibodies to focal adhesion kinase (FAK) and Lamin A/C (H-110) were from Santa Cruz Biotechnology, Inc. (Santa Cruz, CA). Actinomycin D and SB203580 (p38 Inhibitor) were obtained from Enzo Life Sciences (Farmingdale, NY).

### Preparation of Recombinant Fibronectin Modules

His-tagged recombinant fibronectin modules, FnIII-1c, FnIII-13 and FnIII-10n were prepared as previously described [27]. EDA cDNA, a gift from Dr. Livingston Van de Water (Albany Medical College, Albany, NY) [14], was inserted into bacterial expression vector pQE-30 in-frame with an N-terminal 6x His Tag (Qiagen, Inc., Valencia, CA). cDNA was then transformed into competent M15 bacteria. EDA was expressed and purified by affinity chromatography using metal-chelating nitrilotriacetic acid-agarose (Ni-NTA) (Qiagen). EDA recombinant proteins were further purified by passage through a Sephadex G-25 column (GE Healthcare Life Sciences, Pittsburgh, PA), followed by anion-exchange chromatography using a DEAE Sepharose column (GE Healthcare). The purity was assessed by appearance of a single band on SDS-PAGE. All recombinant proteins were assayed for the presence of contaminating endotoxin (<0.25 units/nmol of protein) using the limulus amoebocyte lysate assay, QCL-1000 (Lonza, Allendale, NJ).

### Cell Treatment and Lysis

Human dermal fibroblasts in Dulbecco's Modified Eagle medium (DMEM; Life Technologies, Grand Island, NY) containing 10% FBS (HyClone Laboratories, Logan, UT) were grown to confluence and then maintained at 37°C in an atmosphere of 8% CO_2_. THP-1 cells were purchased from ATCC and maintained in Roswell Park Memorial Institute Media (RPMI-1640) containing 10% FBS, 0.05 mM 2-mercaptoethanol at 37°C in an atmosphere of 5% CO_2_. Prior to use in experiments, fibroblast monolayers and THP-1 cells were serum-starved in DMEM or RPMI with 0.1% BSA, respectively for 24 h. When pharmacological inhibitors were used, cells were pre-treated with inhibitor for 1 h prior to treatment with matrix molecules. Specific treatment times and amounts are provided in the Figure legends. For analysis of whole cell lysates, cells were placed on ice post-treatment, washed once in PBS and lysed in 1X SDS-PAGE sample buffer (62.5 mM Tris, pH = 6.8, 2% SDS, 10% Glycerol, and 5% β-mercaptoethanol) supplemented with Complete Protease Inhibitor (Roche Applied Science, Indianapolis, IN). Protein concentrations were assayed with a Bicinchoninic acid protein reagent kit (Thermo Scientific, Rockford, IL) using BSA as a standard monocyte.

### Nuclear Protein Extraction

For analysis of nuclear extracts cells were lysed in 10 mM HEPES, pH 7.9, 5 mM KCl, 1.5 mM MgCl_2_, 1 mM NaF, 1 mM NA_3_VO_4_, and 0.1% Nonidet P-40. Cell lysates were passed through a 24-gauge needle 3 times and centrifuged at 4°C, 3000×g for 5 min. The nuclear pellet was washed with lysis buffer and nuclear proteins were extracted using a high salt buffer (20 mM HEPES, 400 mM NaCl, 1.5 mM MgCl2, 1 mM NaF, 1 mM Na3VO4, and 10% glycerol, pH 7.9). Nuclear lysates were clarified by centrifugation at 20,000×g for 15 min at 4°C.

### Immunoblot Analysis

Samples were subjected to gel electrophoresis on SDS-polyacrylamide gels under reducing conditions and were transferred onto nitrocellulose membranes (GE Healthcare). For further detection of loading control, membranes were blocked in TBST (Tris-HCL, pH = 7.4, 150 mM NaCl and 0.1% Tween 20) containing 5% w/v BSA and incubated with primary antibodies overnight at 4°C. Secondary antibodies conjugated with horseradish peroxidase were used and detected by enhanced chemiluminescence reagent (GE Healthcare). Membranes were stripped in 62.5 mM Tris-HCl (pH = 6.8), 2% SDS, and 1% β-mercaptoethanol for 20 min at 60°C to remove previously bound antibodies.

### Analysis of IL-8 mRNA Stability

Cells were stimulated with 20 µM FnIII-1c for 2 h. 10 µg/ml actinomycin D (Enzo Life Sciences) was added to stop further transcription in the presence or absence of 20 µM MK-2 Inhibitor III. Total RNA was extracted with RNeasy extraction kit (Qiagen) at 0, 30 and 60 min after treatment with actinomycin D. The integrity and purity of the RNA was assessed by denaturing agarose gel electrophoresis (clean and sharp 28S, 18S and 5S bands), as well as spectrophotometry via Nanodrop. 1.5 µg of RNA from each sample was reverse-transcribed using the RT^2^ First Strand Kit (Qiagen) according to the manufacturer's instructions. IL-8 and β-actin RT^2^ qPCR Primer Assays (Qiagen) were utilized for cDNA amplification. Quantitative PCR was performed using RT^2^ SYBR Green Mastermix (Qiagen) in a MyiQ Cycler System (Bio-Rad Laboratories). The 2∧^−ΔΔCt^ method was utilized to measure the relative expression levels of IL-8 and β-actin. The results take into account the values for six separate experiments.

### Gene Profiling

Human dermal fibroblasts were grown overnight in complete medium and serum-starved for 24 h prior to treatment with fibronectin modules as previously described [23]. The total RNA was isolated from fibroblasts using RNeasy extraction kit (Qiagen). An RT^2^ First Strand kit (Qiagen) was used to convert 1.5 µg of RNA into cDNA. The cDNA was applied to the Human Inflammatory Response & Autoimmunity PCR Microarray (Qiagen). A MyiQ cycler system (Bio-Rad Laboratories) was utilized for real-time PCR detection. Gene expression profiling was analyzed using an Excel-based PCR array data analysis template provided by the manufacturer. Gene profiling for each sample was done in triplicate. The expression profile shown is the result of one representative experiment.

### Human IL-8 Enzyme-Linked Immunosorbent Assay

Human dermal fibroblasts were cultured in 48-well culture plates until 90% confluent. Cells were serum-starved 24 h prior to treatment with fibronectin modules. When pharmacological inhibitors or blocking antibodies were used, cells were pre-treated for 1 h. Following exposure to fibronectin modules 0, 4, 24 or 48 h, cell conditioned medium was collected and analyzed for IL-8 protein expression using a commercially available human ELISA kit (BD Biosciences, San Diego, CA), as directed by the manufacturer.

## Results

### FnIII-1c regulates IL-8 expression in dermal fibroblasts through TLR4 dependent p38 MAPK/MK-2 signaling

**Figure 1:**
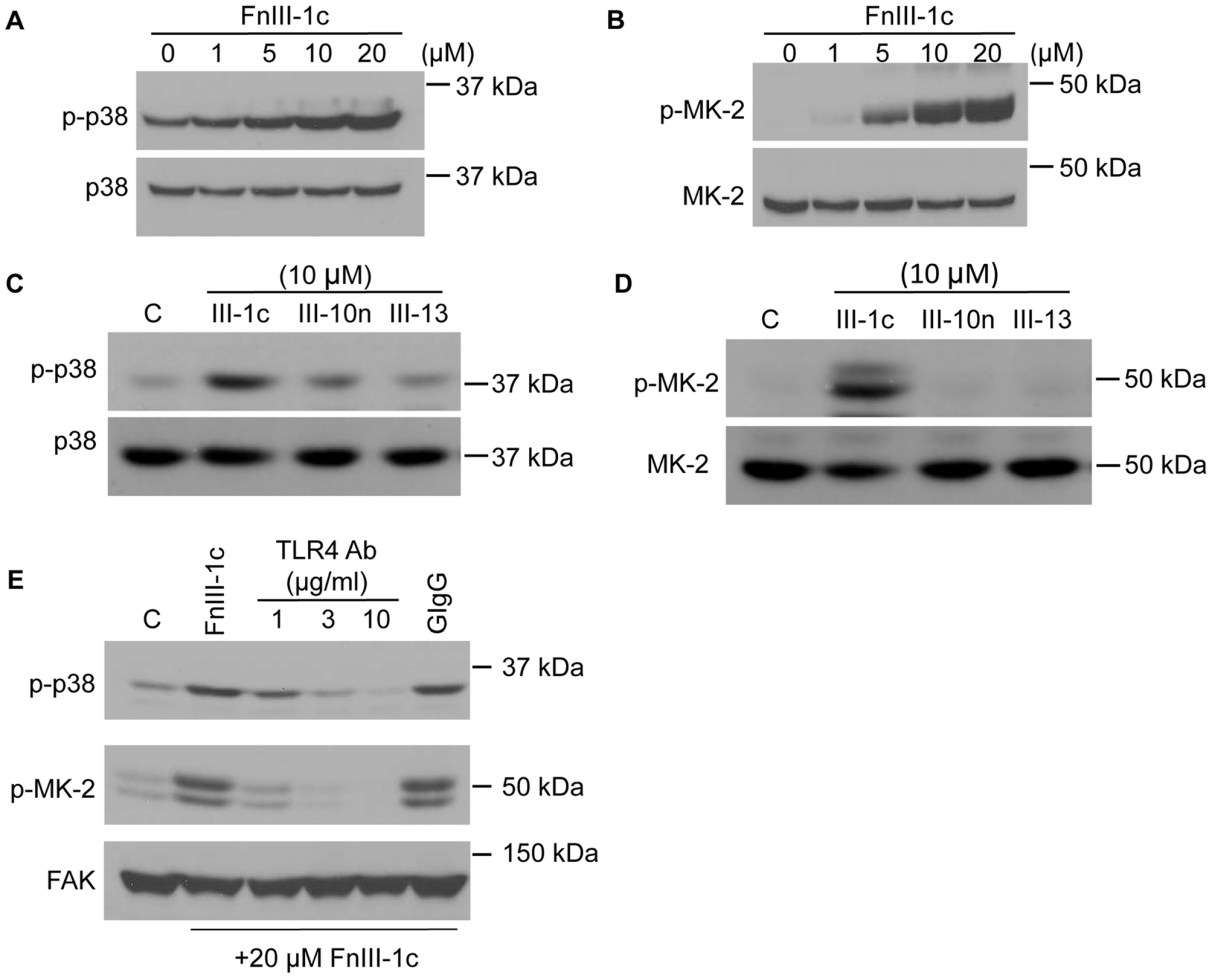
FnIII-1c activates TLR4-dependent p38 MAPK/MK-2 signaling in dermal fibroblasts. Fibroblasts were serum-starved overnight and treated with the indicated amounts of Fn Type III domains, FnIII-1c, FnIII-10n or FnIII-13 for 1 h. Cell layers were lysed and proteins were electrophoresed and immunoblotted with antibodies to phospho-p38 (A, C) and phospho-MK-2 (B, D). Cells were pretreated with increasing concentrations of antibodies to human TLR4 or 20 µg/ml control antibody, goat IgG (GIgG) for 30 min prior to the addition of 20 µM FnIII-1c for 1 h. Cells were lysed and immunoblotted for phospho-p38 and phospho-MK-2 (E). Immunoblotting for total p38, MK-2 or FAK served as loading controls.

Our previous studies have indicated that a fibronectin peptide representing a mechanically unfolded stable intermediate structure of the first Type III domain (FnIII-1c) induces the expression of inflammatory cytokines in human dermal fibroblasts. IL-8 was a major cytokine induced in these cells in response to FnIII-1c and its expression was dependent on the TLR4 dependent activation of NF-κB [23]. Additionally, we have shown that FnIII-1c also activates p38 MAP kinase in fibroblasts [27], [29]. Therefore, experiments were done to determine the role of the p38 MAP kinase signaling in the induction of cytokine expression in dermal fibroblasts by FnIII-1c. The addition of FnIII-1c to human dermal fibroblasts resulted in an increase in phosphorylation of p38 MAP kinase. Activation of p38 MAP kinase by FnIII-1c was dose-dependent and could be detected at the 1–5 µM dose range (Fig. 1A). MAPKAP kinase 2 (MK-2) is a downstream effector of p38 which has been shown to regulate the expression of cytokines in several models of inflammation [30]. We, therefore, evaluated whether MK2 was being activated in human dermal fibroblasts in response to the addition of FnIII-1c. As shown in Fig. 1B, phosphorylation of MK2 was seen in response to FnIII-1c. The effects on MK2 phosphorylation were dose-dependent and occurred over the same concentration range as that seen for FnIII-1c dependent p38 activation (compare with Fig. 1A). Control experiments (Fig. 1C and 1D) indicated that activation of p38 and MK-2 was specific to FnIII-1c as treatment of fibroblasts with other Fn Type III domains (FnIII-10n, FnIII-13) did not result in the phosphorylation of either p38 MAPK or MK-2. These particular Fn domains were selected as controls because they shared characteristics of the FnIII-1c domain, i.e., heparin binding activity (FnIII-13), or a stable inter-mediate structure obtained by mechanical unfolding (FnIII-10n) [31], [32]. To determine whether p38/MK-2 activation by FnIII-1c was dependent on TLR4, blocking antibodies were used to prevent TLR4 signaling. Western blot analysis of FnIII-1c treated fibroblasts showed that phosphorylation of both p38 and MK-2 was completely inhibited when cells were preincubated with blocking antibody to TLR4 (Fig. 1E).

**Figure 2:**
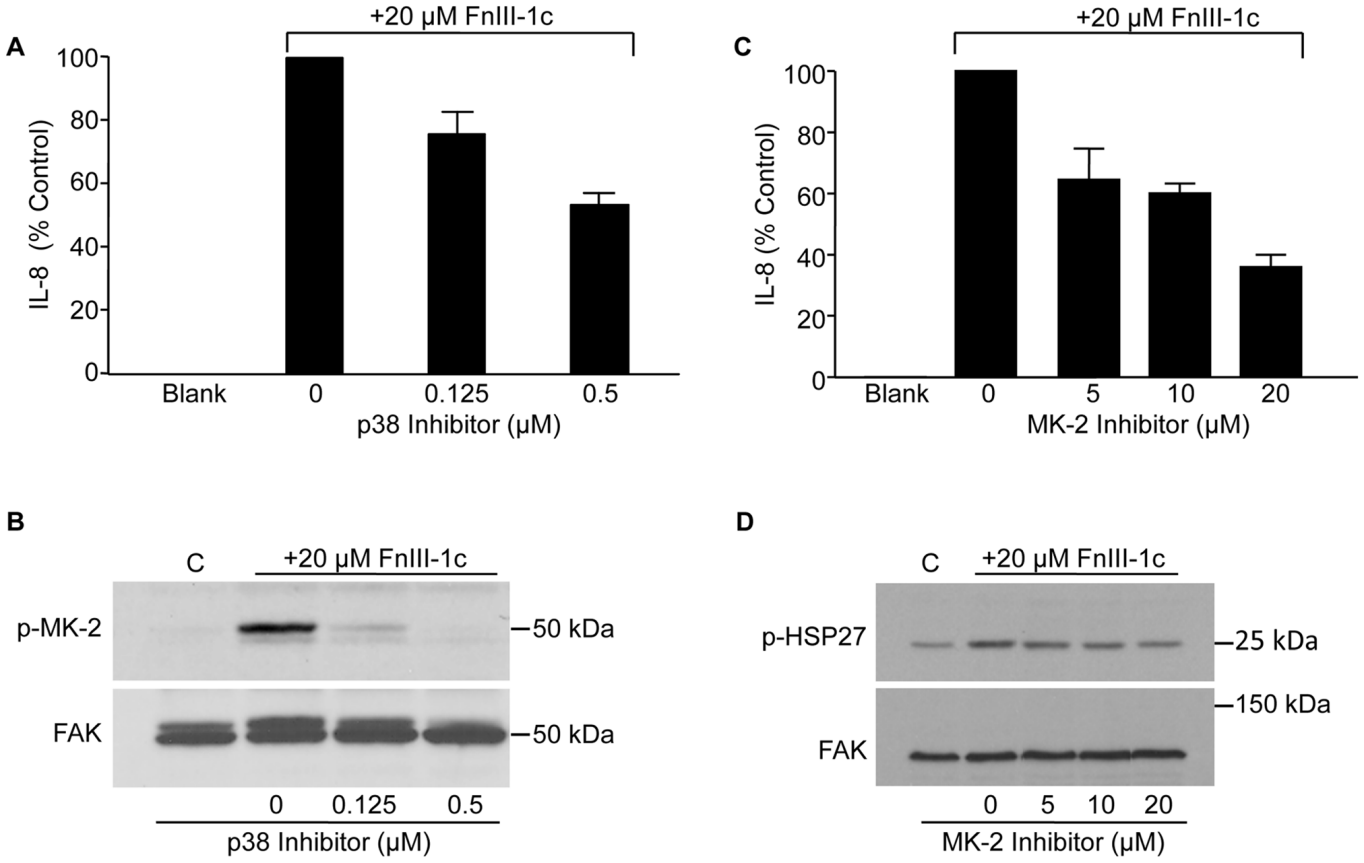
The p38 MAPK/MK-2 pathway regulates FnIII-1c induced IL-8 expression. Fibroblasts were treated with the indicated concentration of inhibitors for p38 (A, B) or MK-2 (C, D) for 1 h prior to stimulation with 20 µM FnIII-1c. The amount of IL-8 protein present in the medium was measured by ELISA at 4 h (A, C). MK-2 phosphorylation in the presence of increasing amounts of p38 inhibitor (SB203580) was assessed using antibodies to phospho-MK-2 (B). HSP27 phosphorylation in the presence of MK-2 inhibitor was assessed by immunoblotting with antibodies to phospho-HSP27. FAK served as loading control (D). Data are expressed as % control where cells which received 20 µM FnIII-1c without inhibitor (0) in A and C are set at 100%. Error bars indicate mean ± SD of 3 separate experiments.

To determine whether the p38/MK2 pathway was involved in the regulation of IL-8 expression in response to FnIII-1c, cells were pre-treated with increasing concentrations of the p38 MAPK inhibitor, SB203580. Treatment with the p38 inhibitor partially attenuated IL-8 expression in response to FnIII-1c (Fig. 2A). Western blot analysis showed that although 0.5 µM SB203580 was sufficient to completely inhibit FnIII-1c induced phosphorylation of MK-2 (Fig. 2B), its inhibitory effect on IL-8 expression was only partial. A similar response was seen with the MK-2 inhibitor which partially blocked IL-8 expression by FnIII-1c (Fig. 2C), even under conditions where MK-2 was completely inhibited from activating its downstream effector, HSP27 (Fig. 2D). Consistent with our previous findings [23], the inhibitors of NFκB activation, PS-1145 and BAY-11-7082 completely prevented the secretion of IL-8 in response to FnIII-1c [23]. Combining inhibitors at suboptimal amounts did not increase the level of inhibition beyond that seen with each inhibitor alone (data not shown). These findings suggest that MK-2 and p38 are part of a signaling axis which functions to modulate the NFκB dependent expression of IL-8.

**Figure 3:**
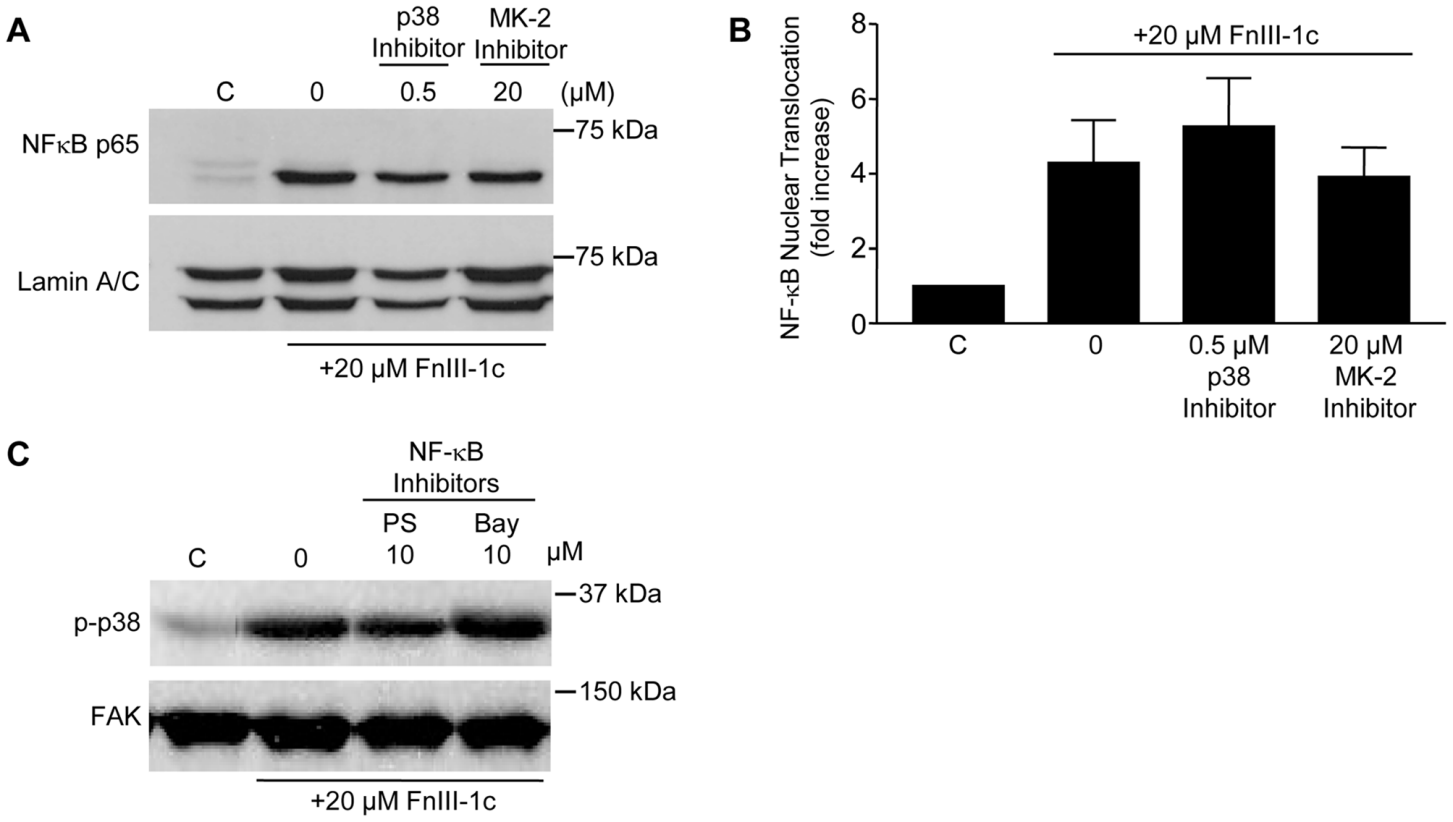
FnIII-1c activates NF-κB in parallel with the p38 MAPK signaling pathway. Monolayers of human fibroblasts were serum-starved overnight and treated with the indicated concentration of p38 inhibitor (SB203580), MK-2 inhibitor (MK-2 inhibitor III), or NFκB inhibitors (PS-1145, Bay11-7082) for 1 h. Cells were then stimulated with 20 µM FnIII-1c for 1vh. Cells were lysed and the nuclear fraction isolated and analyzed by Western blot for the presence of NF-κB protein p65/rel A (A). Blots were quantified by densitometry and normalized to total lamin A/C. Values are mean ± S.D. of 3 separate experiments (B). Cytoplasmic fractions were analyzed by Western blot for p-p38 (C). Membranes were stripped and reprobed with an antibody to nuclear Lamin A/C or FAK, which served as loading control. Proposed signaling pathway (D).

To better understand the relationship between the p38/MK2 and the NF-κB pathways on the FnIII-1c mediated induction of IL-8, experiments were done to determine whether p38 or MK2 regulated the activation of NF-κB. Activation of the NF-κB transcription complex occurs in the cytoplasm and can be characterized by the translocation of the p65/rel A subunit to the nucleus. To determine whether p38 and/or MK-2 play a role in the nuclear translocation of NF-κB by FnIII-1c, cells were treated with FnIII-1c in the presence of the inhibitors to p38 or MK-2 and nuclear extracts were analyzed by Western blot for the presence of p65/rel A. As shown in Figs. 3A and B, FnIII-1c treatment resulted in an increase in nuclear p65/rel A which was unaffected by either the p38 or MK-2 inhibitor indicating that the inhibitory effects of these molecules on IL-8 production did not result from an inhibition of NF-κB activation. Similarly, two inhibitors of NF-κB signaling, PS-1145 and Bay11-8072, were ineffective in blocking FnIII-1c induced activation of p38 (Fig. 3C). These data indicate that the p38 MAPK and NF-κB signaling pathways are activated in parallel by TLR4 in response to FnIII-1c and that they likely function independently to regulate IL-8 expression.

### Activation of MK-2 by FnIII-1c increases IL-8 mRNA stability

**Figure 4:**
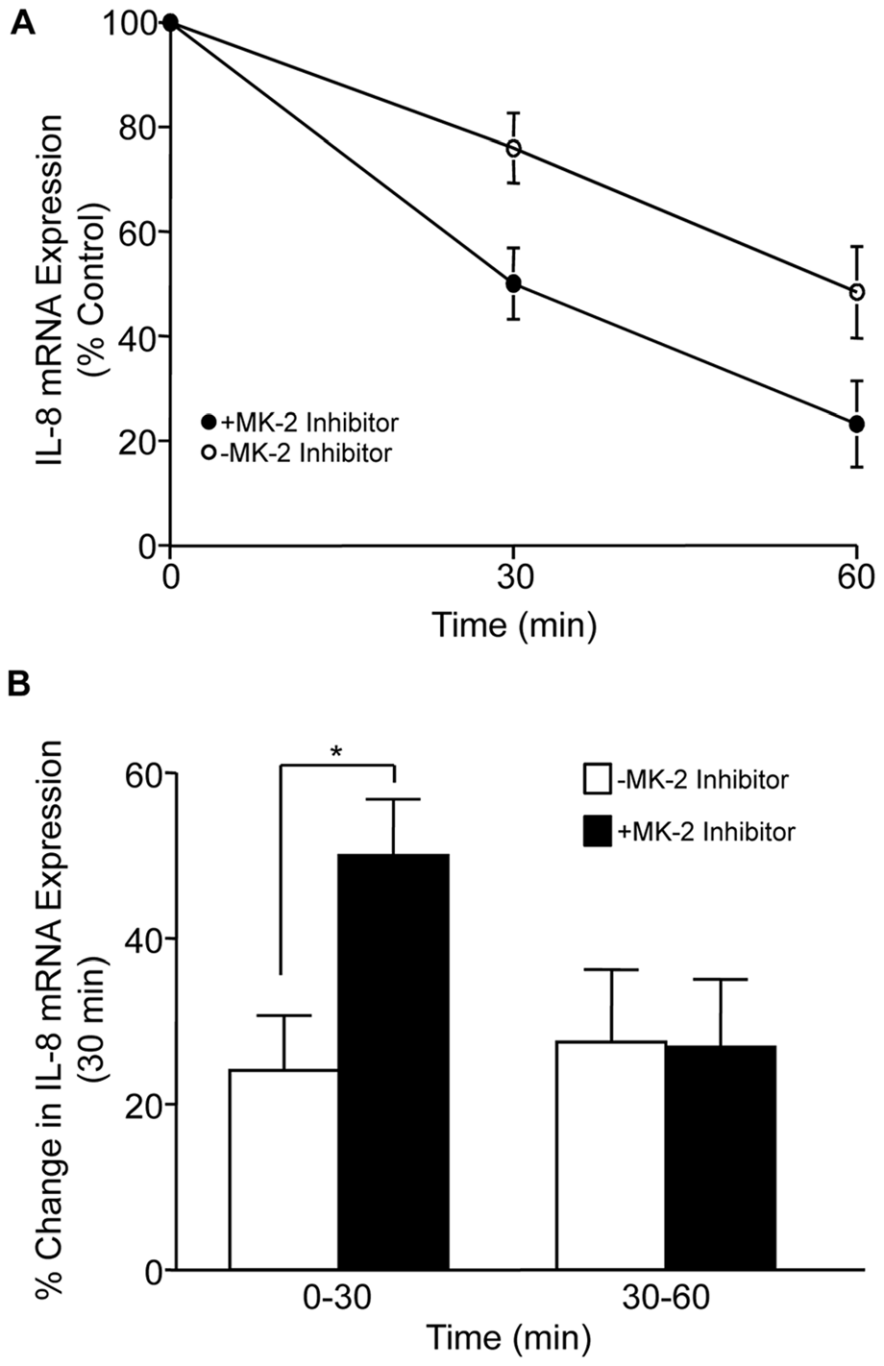
Activation of MK-2 by FnIII-1c regulates IL-8 mRNA stability. Monolayers of dermal fibroblasts were serum-starved overnight and incubated with 10 µM III-1c for 2 h prior to the addition of 10 µM actinomyosin D with or without 20 µM MK-2 inhibitor. After incubation for the indicated times, IL-8 mRNA levels were assessed by RT-PCR. The mRNA level was analyzed relative to the blank control and balanced with the housekeeping gene, β-actin using ΔΔCt (A). The percent (%) change in mRNA expression between 0–30 min and 30–60 min was calculated and analyzed using a two way ANOVA followed by t-test with Bonferroni correction (p<0.05) (B). Error bars indicate mean ± SD of 12 samples. Data are combined from 6 experiments performed in duplicate.

Enhancing cytokine mRNA stability is a mechanism of increasing the amplitude and strength of an inflammatory response. The p38/MK2 pathway has been implicated in the regulation of IL-8 message stability in HeLa cells [33]; therefore, we evaluated whether activation of MK-2 by FnIII-1c affected IL-8 mRNA stability in dermal fibroblasts. To evaluate this question, cells were incubated with FnIII-1c for 2 h to induce IL-8 expression and then treated with actinomycin-D in the presence or absence of the inhibitor of MK2. IL-8 mRNA levels were analyzed by qPCR at 0, 30 and 60 min after the addition of actinomycin. Fig. 4A shows that following treatment with actinomyosin D, IL-8 mRNA levels decreased markedly within an hour. At both the 30 and 60 min time points, cells treated with the MK-2 inhibitor exhibited less IL-8 mRNA than did the untreated controls. Analysis of the data at 30 min intervals (Fig. 4B) showed that the initial rate of decay (0–30 min) was twice as fast in cells which were treated with the MK2 inhibitor, suggesting MK-2 activity prolonged the half-life of IL-8 message. MK2 did not appear to influence the message decay rate at later times (30–60 min). These data suggest that the p38/MK-2 pathway stabilizes IL-8 mRNA expression by dampening the initial rates of decay.

### FnIII-1c and FnEDA induce a similar inflammatory signature in dermal fibroblasts

**Figure 5:**
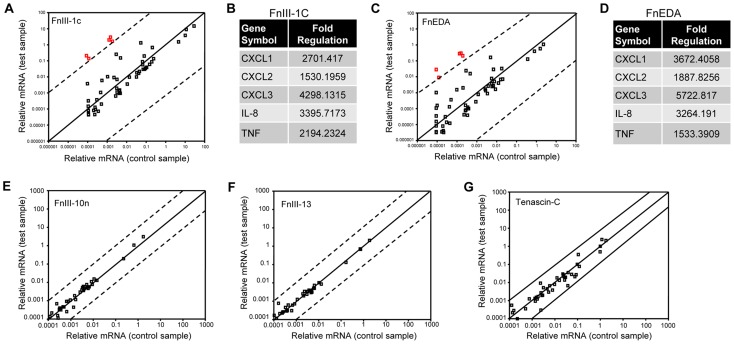
Fibronectin Type III domains: Selective induction of cytokines in fibroblasts. Monolayers of human dermal fibroblasts were serum-starved before treatment with 20 µM FnIII-1c (A), FnEDA (C), FnIII-10n (E), FnIII-13 (F) or 100 µg/ml Tenascin C (G). RNA was extracted and gene expression profiling was performed using a Human Autoimmune and Inflammatory Cytokine PCR array. Dashed lines indicate a 1000 fold change in gene expression (A and C) and a 10 fold increase in gene expression (E, F, G). The 5 genes whose expression was increased ≥1000 fold are shown in tables (B,D).

**Figure 6:**
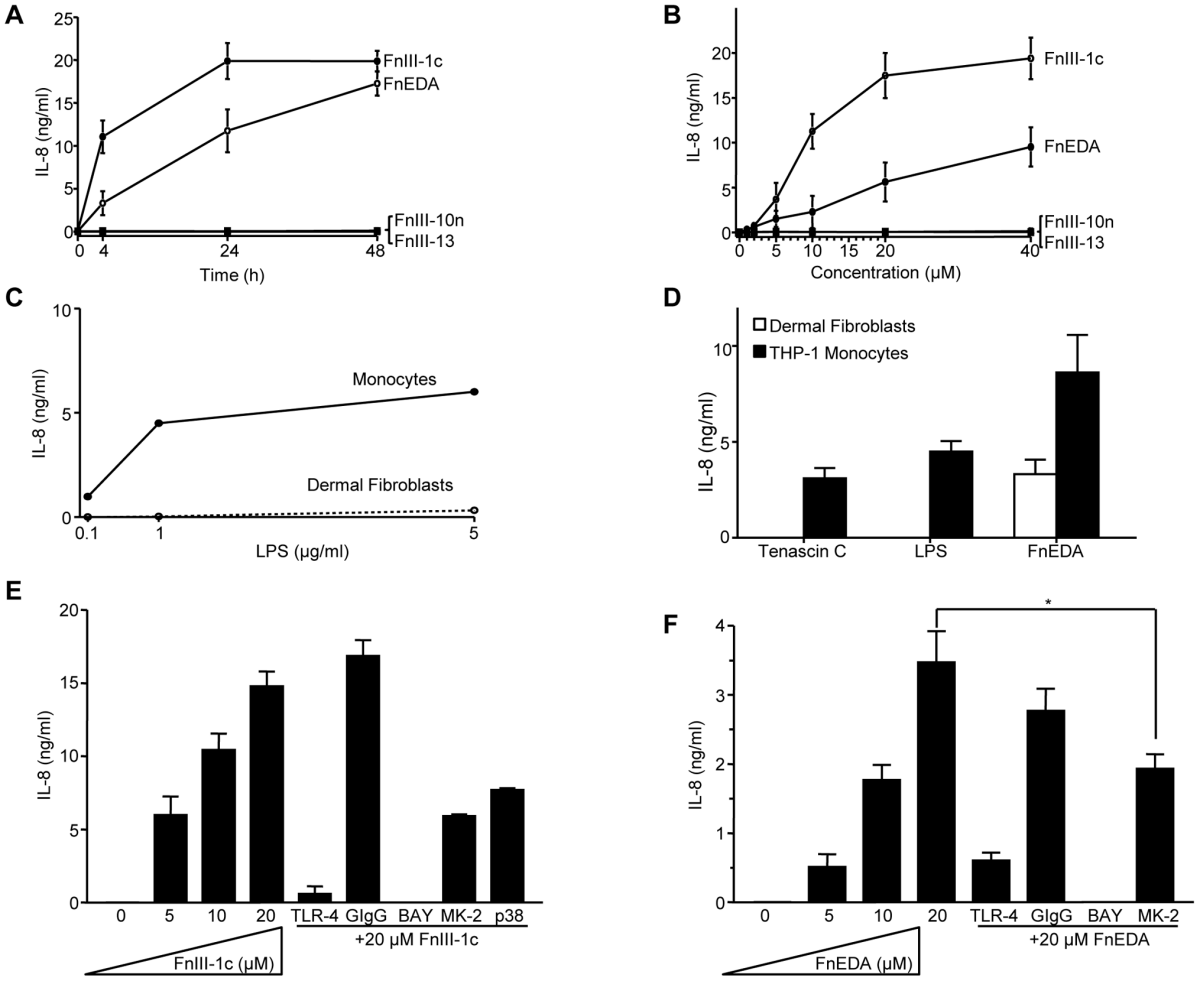
IL-8 expression induced by FnIII domains is NF-κB and TLR4-dependent. Fibroblasts were serum-starved overnight before treatment with 20 µM FnIII-1c, FnEDA, FnIII-10n, FnIII-13 or PBS in 0.1% BSA-DMEM for the indicated amount of time (A) or for 4 h at the indicated dose (B). Fibroblasts or THP-1 monocytes were incubated with increasing doses of LPS for 4 h (C). Tenascin C (100 µg/ml), LPS (1 µg/ml) or FnEDA (200 µg/ml) were incubated with either dermal fibroblasts or THP-1 monocytes for 4 h (D). Cells were pretreated with the indicated amounts of inhibitor or monoclonal antibody for 1 h prior to the addition of FnIII-1c (E) or FnEDA (F) in 0.1% BSA-DMEM for 4 h. In all experiments, conditioned media was collected and IL-8 protein amounts determined by ELISA. To determine significance, statistical analysis was performed using Student's t test. *p<0.05. Error bars indicate mean ± S.D. of three separate experiments performed in triplicate.

Earlier studies have documented a role for the alternatively spliced EDA isoform of fibronectin in the activation of TLR4 signaling and cytokine release in immune cells [20], [21]. Expression of the EDA isoform of fibronectin is restricted to tissues under-going active remodeling, such as during development and wound healing and in association with tissue fibrosis and inflammation. To address whether the EDA domain of fibronectin could also induce the expression of cytokines in dermal fibroblasts, we used the Human Autoimmune and Inflammatory Cytokine microarray to compare the expression profile of inflammatory genes induced in dermal fibroblasts treated individually with these fibronectin domains. Incubation of dermal fibroblasts with FnIII-1c resulted in the increased expression of approximately 20 genes (Fig. 5A). In particular, the expression of 5 genes, chemokine ligand-1, -2 and -3 (CXCL1, CXCL2, CXCL3), Interleukin-8 (IL-8) and tumor necrosis factor-alpha (TNF-α) were increased greater than 1000 fold within 2 hours (Fig. 5B). A similar response was seen using the FnEDA domain (Fig. 5C), where the same 5 genes were selectively increased (Fig. 5D). In contrast, cells incubated with either the FnIII-10n or FnIII-13 showed little change in the expression of any genes (Fig. 5E and F). Tenascin-C is an extracellular matrix protein which is expressed in injured tissues, tumors and fibrotic diseases [reviewed in [34]], where it functions as a DAMP to induce TLR4-dependent cytokine expression in synovial cells [35]. However, Tenascin C was unable to induce cytokine expression in dermal fibroblasts (Fig. 5G), suggesting that dermal fibroblasts selectively respond to fibronectin-derived DAMPs. As shown in Fig. 6A, addition of either FnIII-1c or FnEDA to dermal fibroblasts resulted in an increase in the expression of IL-8. IL-8 accumulated in the medium for several hours in response to FnIII-1c reaching an apparent steady state by 24 h. FnEDA also induced IL-8 expression but with somewhat slower kinetics, however, both domains resulted in similar concentrations of IL-8 in the conditioned medium by 48 h (18–20 ng/ml). The effects of both FnEDA and FnIII-1c on IL-8 concentration was dose dependent (Fig. 6B). The difference in the relative amounts of IL-8 induced by each module reflects differences in the rates of accumulation in the medium at the 4-h time point (see Fig. 6A). Neither FnIII-10n nor FnIII-13 induced IL-8 expression.

Bacterial lipopolysaccharide (LPS) is the canonical ligand for TLR4. It is a well established activator of TLR4-dependent cytokine release in immune cells (reviewed in [36]). However, when added to dermal fibroblasts, LPS was unable to induce IL-8 expression in dermal fibroblasts (Fig. 6C). As expected, both Tenascin C and LPS induced IL-8 expression in the monocytic cell line, THP-1 (Fig. 6C and D). These data suggest that the induction of an inflammatory response by TLR4 ligands is cell-type specific.

To determine whether the NF-κB and the p38/MK-2 signaling pathways play a role in IL-8 induction in response to FnEDA, cells were incubated with FnEDA in the presence of various inhibitors of the TLR4 signaling pathway. As shown in Fig. 6E and F, induction of IL-8 in response to either FnIII-1c or FnEDA was completely inhibited by blocking antibody to TLR4 consistent with FnEDA and FnIII-1c both working through TLR4 to induce cytokine expression. The inhibitor of the NF-κB pathway (BAY) completely blocked IL-8 expression indicating that gene expression in response to either FnIII-1c or FnEDA was regulated by NF-κB. The inhibitor of p38 or its down-stream effector, MK-2, only partially blocked the induction of IL-8 by either FnIII-1c or FnEDA. These data suggest that both the FnEDA and FnIII-1c work through NF-κB and p38/MK-2 signaling to induce an inflammatory response in dermal fibroblasts.

### FnEDA-enhancement of FnIII-1c induced IL-8 release

**Figure 7:**
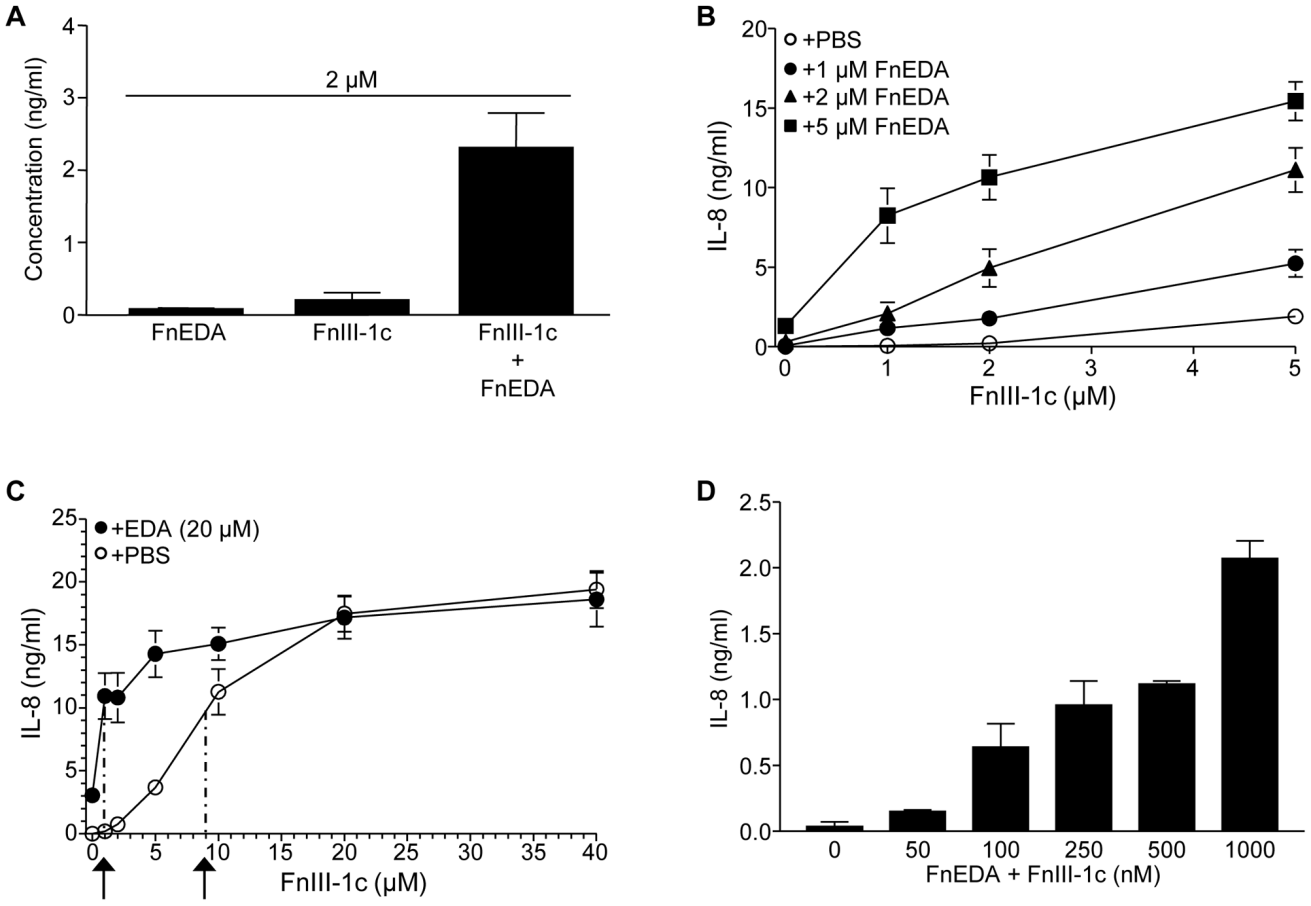
FnEDA and FnIII-1c domains work synergistically to regulate IL-8 expression. Monolayers of human dermal fibroblasts were serum-starved overnight and then incubated with FnIII-1c and FnEDA alone or in combination. After 4 h (A, B, C) or 24 h (D), the amount of IL-8 protein in the conditioned medium was determined by ELISA. Dashed lines and arrows in (C) indicate the approximate Km of FnIII-1c on IL-8 production. Specific amounts of each module are provided in the Figure. Error bars indicate mean ± S.D. of three experiments performed in triplicate.

Our data demonstrate that FnIII-1c and FnEDA induce the expression of IL-8 through the same molecular mechanism in dermal fibroblasts. Activation of the p38 MAPK and NF-κB signaling pathways, as well as induction of IL-8, was shown to be dependent on TLR4 activity. Enhancement of TLR4 activation as a result of ligand sensitizing molecules has been well documented in a variety of immune cells [37]. Here we evaluated the combined effect of the FnEDA and FnIII-1c domains on IL-8 expression. FnEDA and FnIII-1c were added to cells in amounts well below saturation. As shown in Fig.7A, when added individually at 2 µM, FnIII-1c and FnEDA respectively induced 100 or 200 ng/ml IL-8 over a 4-h period. However, when both modules were added simultaneously, IL-8 induction was increased to 2.5 ng/ml. This amount is 8 fold greater than the expected additive effect of 0.3 ng/ml. These results suggest a synergistic effect of these two domains on cytokine expression. To examine this synergism more closely, cells were incubated with increasing concentrations of FnIII-1c in the presence of a fixed amount of FnEDA. As shown in Fig. 7B, when cells were incubated with FnEDA at amounts low enough to yield little detectable IL-8, the production of IL-8 in response to FnIII-1c was enhanced at all doses. When analyzed over the complete dose range of FnIII-1c, the addition of a fixed amount of FnEDA shifted the effective dose curve of FnIII-1c to the left consistent with FnEDA sensitizing the cells to FnIII-1c (Fig. 7C). In the absence of FnEDA, induction of IL-8 by FnIII-1c was half-maximal at approximately 9 µM FnIII-1c. In the presence of FnEDA, half-maximal IL-8 induction was seen at 0.9 µM FnIII-1c, suggesting at 10-fold shift in sensitivity of the cells to FnIII-1c. These experiments used µM concentrations of fibronectin peptides in short-term (4 h) incubations to generate relatively large amounts of IL-8 (5–20 ng/ml). High affinity receptors for IL-8 can be activated at pM concentrations of IL-8 [38]. To determine the minimal amount of peptide required to induce pM amounts of IL-8, peptides were combined at nM concentrations and incubated with cells for 24 h. Figure 7D shows that the FnIII-1c and FnEDA could generate an IL-8 response at doses between 50–100 nM.

## Discussion

Previous experiments have demonstrated that alterations to fibronectin structure, both biochemical and mechanical, occur during periods of active ECM remodeling. Emerging data indicate that alterations to fibronectin structure (i.e., inclusion of alternatively spliced EDA domain) can lead to activation of innate immune responses via TLRs [22], [23], [39]. The present study demonstrates that two Type III domains from fibronectin, one regulated by mechanical force and proteolysis (FnIII-1) and a second regulated by alternative splicing of mRNA (FnEDA), can induce pro-inflammatory cytokine expression through NF-κB-dependent gene transcription and subsequent mRNA stability controlled by the p38-MK-2 signaling axis. Activation of the p38-MK-2 signaling axis and induction of IL-8 gene expression, by both FnIII-1c and FnEDA, was shown to be dependent on TLR4 indicating a link between the fibroblasts responsible for the remodeling of the ECM and the innate immune system.

Both FnIII-1c and FnEDA induced a similar pro-inflammatory gene signature in human dermal fibroblasts. CXCL-1,-2,-3, TNF-alpha and IL-8 were the most highly upregulated cytokines. Typically in a normal tissue microenvironment there are low levels of these pro-inflammatory mediators, as their expression is tightly regulated by a variety of molecular mechanisms. In part, this is due to the fact that the mRNA of pro-inflammatory cytokines is extremely short-lived and rarely becomes translated into protein as a result of the prominence of AU-rich elements in the 3′-untranslated region. However, when damage or pathogenic infection occurs, the cytokine mRNAs become rapidly stabilized, resulting in an innate immune response mounted by stromal components of the tissue [40]. Upon stimulation of the TLR family, downstream signaling pathways, such as p38 MAP Kinase, are activated to prolong mRNA stability [41], [42]. More specifically, the downstream substrate of p38 MAPK, MK-2, can phosphorylate and inactivate AU-binding proteins, thereby stabilizing cytokine mRNA expression by dampening the initial rates of decay [43], [44]. These signaling pathways are known to be activated downstream of TLR4 in a variety of immune cells [45], [46]. We have now demonstrated that this signaling axis controls cytokine expression in dermal fibroblasts in response to TLR4 activation. Our data also identified FnIII-1c and FnEDA as novel activators of p38/MK-2 signaling, ultimately leading to the enhanced stabilization of IL-8 mRNA.

Pharmacological inhibition of the p38 MAPK pathway has long been studied as a potential therapeutic avenue to promote anti-inflammatory effects in rheumatoid arthritis, cirrhosis, fibrosis and cancer [47]. However, the p38 MAPK inhibitors are ATP competitors that can cross-react with other kinases, resulting in detrimental side-effects [48] and alternative mechanisms to block aberrant signaling of p38 MAPK/MK-2 need to be investigated. Targeting mechanisms regulating fibronectin secondary structure (i.e., mechanical unfolding, proteolysis) or alternative splicing (EDA^+^ fibronectin) may prove to be an effective alternative to dampen inflammation within a fibronectin-rich microenvironment.

A wide range of ECM-derived DAMPs have been identified as activators of TLR4 signaling in a variety of cell types [49]. MD-2, myeloid differentiation factor-2, is the LPS-binding constituent of the TLR4 receptor complex and it is often required for activation of downstream signaling pathways [50], [51]. The ligand specificity of TLR4 signaling, however, can be regulated through distinct ancillary proteins or co-receptors, such as CD14, CD44 and CD36, which are responsible for ligand delivery to the TLR4/MD-2 complex [35], [52], [53]. Whether these distinct receptor complexes function on the same cell to regulate differential TLR4 signaling in response to different ligands has not been demonstrated. Furthermore, it is not clear whether co-receptors are responsible for differential gene expression in response to a single ligand. For example, Midwood, et al. [35] demonstrated that Tenascin C induced the TLR4 dependent expression of IL-6 in synovial fibroblasts. In contrast, Tenascin C elicited a more elaborate cytokine profile in macrophages: IL-6, IL-8 and TNF-α. In our studies, LPS and Tenascin-C did not elicit an inflammatory response in dermal fibroblasts, but did induce an IL-8 response in monocytic cells. This suggests that dermal fibroblasts may not express co-receptors required to activate TLR4 signaling by LPS or Tenascin C. The ability of dermal fibroblasts to respond to fibronectin Type III domains indicates that the co-receptors required for TLR4 activation, in response to Tenascin C may be different from those required by FnIII-1c or FnEDA. These data suggest that heterogeneity of TLR-4 containing receptor complexes may regulate the cellular response to individual ligands thereby providing a mechanism by which cells can selectively regulate their response to individual TLR4 activators. The existence of receptor complexes which can differentially regulate the cellular response to DAMPs and PAMPs suggests that it is possible to design therapeutics to target chronic inflammation without sacrificing host defense.

The physiological conditions under which the FnIII-1 and FnEDA domains activate inflammatory pathways are not well understood. The fibronectin matrix is a highly dynamic structure that undergoes continuous assembly and turnover in most tissue. Mechanically regulated cryptic sites, proteolysis and changes in alternative splicing of matrix fibronectin are emerging as influential events in the acquisition of disease-associated phenotypes [54]. Proteomic analysis has indicated that both the EDA domain and the III-1c peptide could be released from the matrix by MMP2 [26]. Although the presence of fibronectin fragments has been documented in injured and disease tissue [6], [55]–[57], little is known about their specific sequences and concentrations within the local microenvironment. Force-induced unfolding of the FnIII-1 domain has been shown to expose cryptic activity regulating fibronectin polymerization [9], [58], [59], cell growth [60], [61] and skeletal muscle contraction [60], [61]. However, little is known about the contribution of these unfolded Type III domains to the inflammatory response. Interestingly, fibronectin has been linked to mechanically-induced activation of NFκB and IL-8 induction in endothelial cells and amniotic epithelium [62], [63], consistent with the concept that force-induced unfolding of Type III domains within fibronectin may contribute to cytokine release under physiological conditions. Recently, UV absorption microscopy has estimated the concentration of fibronectin in a polymerized fibronectin fiber at 177 mg/ml suggesting a local concentration of fibronectin of up to 400 µM [64]. Limited fiber proteolysis would therefore be expected to generate the levels of Fn peptides required for a robust induction of IL-8.

In the adult, matrix fibronectin is constantly replenished by fibronectin from the plasma [65]–[68]. Plasma fibronectin is synthesized by the liver and does not contain the EDA domain. Resident tissue cells, primarily fibroblasts, polymerize plasma fibronectin into the stromal matrix; however, in response to injury or during disease processes, resident cells synthesize cellular fibronectin which is alternatively spliced to include the extra domains, EDA and EDB. The EDA domain is considered a marker for fibrosis [69] and neovascular metastasis [70]. EDA-mediated TLR4 signaling is thought to contribute to adverse inflammation following cardiac infarction [71], cerebral ischemia [72], allergen challenge [73], graft vs. host disease [74], and pre-term birth [75]. Our data provide the first evidence that biochemical and/or mechanical alterations in the topography of the fibronectin matrix may work coordinately and synergistically to maximize the tissue response to damage. While both the FnEDA and FnIII-1c domains induce identical cytokine profiles through the same signaling pathways, their effects on cytokine expression are synergistic rather than additive. This synergy suggests unshared but interactive aspects of their individual mechanisms of action. Our data further suggest that alternative splicing of fibronectin to include the EDA domain may act as a priming event to sensitize cells to respond more rapidly to matrix damage following mechanical or proteolytic insult and point to the organization and composition of the fibronectin matrix as a previously unrecognized target for the control of chronic tissue inflammation.

## References

[1] Bradshaw MJ, Smith ML (2013) Multiscale relationships between fibronectin structure and functional properties. Acta Biomater. Available: http://dx.doi.org/10.1016/j.actbio.2013.08.027. 10.1016/j.actbio.2013.08.027 23978411

[2] Peleg O, Savin T, Kolmakov GV, Salib IG, Balazs AC, et al (2012) Fibers with integrated mechanochemical switches: minimalistic design principles derived from fibronectin. Biophys J 103: 1909–1918. 23199919 10.1016/j.bpj.2012.09.028 PMC3491717

[3] Muro AF, Iaconcig A, Baralle FE (1998) Regulation of the fibronectin EDA exon alternative splicing. Cooperative role of the exonic enhancer element and the 5′ splicing site. FEBS Lett 437: 137–141. 9804187 10.1016/s0014-5793(98)01201-0

[4] Watanabe K, Takahashi H, Habu Y, Kamiya-Kubushiro N, Kamiya S, et al (2000) Interaction with heparin and matrix metalloproteinase 2 cleavage expose a cryptic anti-adhesive site of fibronectin. Biochemistry 39: 7138–7144. 10852711 10.1021/bi992670r

[5] Schedin P, Strange R, Mitrenga T, Wolfe P, Kaeck M (2000) Fibronectin fragments induce MMP activity in mouse mammary epithelial cells: evidence for a role in mammary tissue remodeling. J Cell Sci 113: 795–806. 10671369 10.1242/jcs.113.5.795

[6] Sofat N (2009) Analysing the role of endogenous matrix molecules in the development of osteoarthritis. Int J Exp Pathol 90: 463–479. 19765101 10.1111/j.1365-2613.2009.00676.x PMC2768145

[7] Smith ML, Gourdon D, Little WC, Kubow KE, Eguiluz RA, et al (2007) Force-induced unfolding of fibronectin in the extracellular matrix of living cells. PLoS Biol 5 (e268): 2243–2254. 10.1371/journal.pbio.0050268 PMC1994993 17914904

[8] Hocking DC, Smith RK, McKeown-Longo PJ (1996) A novel role for the integrin-binding III-10 module in fibronectin matrix assembly. J Cell Biol 133: 431–444. 8609174 10.1083/jcb.133.2.431 PMC2120803

[9] Zhong C, Chrzanowska-Wodnicka M, Brown J, Shaub A, Belkin AM, et al (1998) Rho-mediated contractility exposes a cryptic site in fibronectin and induces fibronectin matrix assembly. J Cell Biol 141: 539–551. 9548730 10.1083/jcb.141.2.539 PMC2148448

[10] Gee EP, Yuksel D, Stultz CM, Ingber DE (2013) SLLISWD sequence in the 10FNIII domain initiates fibronectin fibrillogenesis. J Biol Chem 288: 21329–21340. 23740248 10.1074/jbc.M113.462077 PMC3774401

[11] Vakonakis I, Staunton D, Ellis IR, Sarkies P, Flanagan A, et al (2009) Motogenic sites in human fibronectin are masked by long range interactions. J Biol Chem 284: 15668–15675. 19366708 10.1074/jbc.M109.003673 PMC2708863

[12] Krammer A, Craig D, Thomas WE, Schulten K, Vogel V (2002) A structural model for force regulated integrin binding to fibronectin's RGF-synergy site. Matrix Biol 21: 139–147. 11852230 10.1016/s0945-053x(01)00197-4

[13] Mao Y, Schwarzbauer JE (2005) Stimulatory effects of a three-dimensional microenvironment on cell-mediated fibronectin fibrillogenesis. J Cell Sci 118: 4427–4436. 16159961 10.1242/jcs.02566

[14] Shinde AV, Bystroff C, Wang C, Vogelezang MG, Vincent PA, et al (2008) Identification of the peptide sequences within the EIIIA (EDA) segment of fibronectin that mediate integrin a9b1-dependent cellular activities. J Biol Chem 283: 2858–2870. 17967897 10.1074/jbc.M708306200

[15] Miura S, Kamiya S, Saito Y, Wada S, Hayashi R, et al (2007) Antiadhesive sites present in the fibronectin type III-like repeats of human plasma fibronectin. Biol Pharm Bull 30: 891–897. 17473431 10.1248/bpb.30.891

[16] Mitsi M, Forsten-Williams K, Gopalakrishnan M, Nugent MA (2008) A catalytic role of heparin within the extracellular matrix. J Biol Chem 283: 34796–34807. 18845539 10.1074/jbc.M806692200 PMC2596404

[17] Chabria M, Hertig S, Smith ML, Vogel V (2010) Stretching fibronectin fibres disrupts binding of bacterial adhesins by physicially destroying an epitope. Nature Communications 1: 135. 10.1038/ncomms1135 PMC3105298 21139580

[18] O'Neill LA, Bryant CE, Doyle SL (2009) Therapeutic targeting of Toll-like receptors for infectious and inflammatory diseases and cancer. Pharmacol Rev 61: 177–197. 19474110 10.1124/pr.109.001073 PMC2846156

[19] Terhorst D, Kalali BN, Ollert M, Ring J, Mempel M (2010) The role of toll-like receptors in host defenses and their relevance to dermatologic diseases. Am J Clin Dermatol 11: 1–10. 20000870 10.2165/11311110-000000000-00000

[20] Gondokaryono SP, Ushio H, Niyonsaba F, Hara M, Takenaka H, et al (2007) The extra domain A of fibronectin stimulates murine mast cells via toll-like receptor 4. J Leukocyte Biol 82: 657–665. 17575266 10.1189/jlb.1206730

[21] McFadden JP, Basketter DA, Dearman RJ, Kimber IR (2011) Extra domain A-positive fibronectin-positive feedback loops and their association with cutaneous inflammatory disease. Clin Dermatol 29: 257–265. 21496732 10.1016/j.clindermatol.2010.11.003

[22] Zheng M, Jones DM, Horzempa C, Prasad A, McKeown-Longo PM (2011) The first type III domain of fibronectin is associated with the expression of cytokines within the lung tumor microenvironment. J Cancer 2: 478–483. 21980322 10.7150/jca.2.478 PMC3187932

[23] You R, Zheng M, McKeown-Longo PJ (2010) The first type III repeat in fibronectin activates an inflammatory pathway in dermal fibroblasts. J Biol Chem 285: 36255–36259. 20923762 10.1074/jbc.C110.176990 PMC2978552

[24] Gao M, Craig D, Lequin O, Campbell ID, Vogel V, et al (2003) Structure and functional significance of mechanically unfolded fibronectin type III1 intermediates. Proc Natl Acad Sci 100: 14784–14789. 14657397 10.1073/pnas.2334390100 PMC299803

[25] Briknarova K, Akerman ME, Hoyt DW, Ruoslahti E, Ely KR (2003) Anastellin, an FN3 fragment with fibronectin polymerization activity, resembles amyloid fibril precursors. J Mol Biol 332: 205–215. 12946358 10.1016/s0022-2836(03)00890-8

[26] Doucet A, Overall CM (2011) Broad coverage identification of multiple proteolytic cleavage site sequences in complex high molecular weight proteins using quantitative proteomics as a complement to edman sequencing. Mol Cell Proteomics 10: M110.003533. 10.1074/mcp.M110.003533 PMC3098582 20876890

[27] Klein RM, Zheng M, Ambesi A, van de Water L, McKeown-Longo PJ (2003) Stimulation of extracellular matrix remodeling by the first type III repeat in fibronectin. J Cell Sci 116: 4663–4674. 14576359 10.1242/jcs.00778

[28] Chandler EM, Saunders MP, Yoon CJ, Gourdon D, Fishbach C (2011) Adipose progenitor cells increase fibronectin matrix strain and unfolding in breast tumors. Phys Biol 8: 015008-doi:10.1088/1478-3975/8/1/015008. 10.1088/1478-3975/8/1/015008 21301062

[29] You R, Klein RM, Zheng M, McKeown-Longo PJ (2009) Regulation of p38 MAP kinase by anastellin is independent of anastellin's effect on matrix fibronectin. Matrix Biol 28: 101–109. 19379667 10.1016/j.matbio.2009.01.003 PMC2692086

[30] Ronkina N, Kotlyarov A, Gaestel M (2008) MK2 and MK3–a pair of isoenzymes. Front Biosci 13: 5511–5521. 18508601 10.2741/3095

[31] Busby TF, Argraves WS, Brew SA, Pechik I, Gilliland GL, et al (1995) Heparin binding by fibronectin module III-13 involves six discontinuous basic residues brought together to form a cationic cradle. J Biol Chem 270: 18558–18562. 7629186 10.1074/jbc.270.31.18558

[32] Gee EP, Ingber DE, Stultz CM (2008) Fibronectin unfolding revisited: modeling cell traction-mediated unfolding of the tenth type-III repeat. PLoS ONE 3: e2373. 19020673 10.1371/journal.pone.0002373 PMC2585069

[33] Winzen R, Kracht M, Ritter B, Wilhelm A, Chen CY, et al (1999) The p38 MAP kinase pathway signals for cytokine-induced mRNA stabilization via MAP kinase-activated protein kinase 2 and an AU-rich region-targeted mechanism. EMBO J 18: 4969–4980. 10487749 10.1093/emboj/18.18.4969 PMC1171568

[34] Udalova IA, Ruhmann M, Thomson SJ, Midwood KS (2011) Expression and immune function of tenascin-C. Crit Rev Immunol 32: 115–145. 10.1615/critrevimmunol.v31.i2.30 21542790

[35] Midwood K, Sacre S, Piccinini AM, Inglis J, Trebaul A, et al (2009) Tenascin-C is an endogenous activator of Toll-like receptor 4 that is essential for maintaining inflammation in arthritic joint disease. Nature Med 15: 774–780. 19561617 10.1038/nm.1987

[36] Schultz GS, Davidson JM, Kirsner RS, Bornstein P, Herman IM (2011) Dynamic reciprocity in the wound microenvironment. Wound Repair Regen 19: 134–148. 21362080 10.1111/j.1524-475X.2011.00673.x PMC3051353

[37] Erridge C (2010) Endogenous ligands of TLR2 and TLR4: agonists or assistants? J Leukocyte Biol 87: 989–999. 20179153 10.1189/jlb.1209775

[38] Schumacher C, Clark-Lewis I, Baggiolini M, Moser B (1992) High- and low-affinity binding of GROa and neutrophil-activating peptide 2 to interleukin 8 receptors on human neutrophils. Proc Natl Acad Sci USA 89: 10542–10546. 1438244 10.1073/pnas.89.21.10542 PMC50375

[39] Okamura Y, Watari M, Jerud ES, Young DW, Ishizaka ST, et al (2001) The extra domain A of fibronectin activates Toll-like receptor 4. J Biol Chem 276: 10229–10233. 11150311 10.1074/jbc.M100099200

[40] Knapinska AM, Irizarry-Barreto P, Adusumalli S, Andorulakis I, Brewer G (2005) Molecular mechanisms regulating mRNA stability: Physiological and pathological significance. Current Genomics 6: 471–486.

[41] Akira S (2003) Mammalian Toll-like receptors. Curr Opin Immunol 15: 5–11. 12495726 10.1016/s0952-7915(02)00013-4

[42] Bowie A, O'Neill LAJ (2000) The interleukin-1 receptor/Toll-like receptor superfamily: signal generators for pro-inflammatory interleukins and microbial products. J Leuk Biol 67: 508–514. 10.1002/jlb.67.4.508 10770283

[43] Frevel MAE, Bakheet T, Silva AM, Hissong JG, Khabar KSA, et al (2003) p38 Mitogen-activated protein kinase-dependent and -independent signaling of mRNA stability of AU-rich element-containing transcripts. Mol Cell Biol 23: 425–436. 12509443 10.1128/MCB.23.2.425-436.2003 PMC151534

[44] Neininger A, Kontoyiannis D, Kotlyarov A, Winzen R, Eckert R, et al (2002) MK2 targets AU-rich elements and regulates biosynthesis of tumor necrosis factor and interleukin-6 independently at different post-transcriptional levels. J Biol Chem 277: 3065–3068. 11741878 10.1074/jbc.C100685200

[45] An H, Yu Y, Zhang M, Xu H, Qi R, et al (2002) Involvement of ERK, p38 and NF-kB signal transduction in regulation of TLR2, TLR4 and TLR9 gene expression induced by lipopolysaccharide in mouse dendritic cells. Immunology 106: 38–45. 11972630 10.1046/j.1365-2567.2002.01401.x PMC1782697

[46] Bode JG, Ehlting C, Häussinger D (2012) The macrophage response towards LPS and its control through the p38^MAPK^-STAT3 axis. Cell Signal 24: 1185–1194. 22330073 10.1016/j.cellsig.2012.01.018

[47] Kumar P, Amin MA, Harlow LA, Polverini PJ, Koch AE (2003) Src and phosphatidylinositol 3-kinase mediate soluble E-selectin-induced angiogenesis. Blood 101: 3960–3968. 12522014 10.1182/blood-2002-04-1237

[48] Sweeney SE, Firestein GS (2006) Mitogen activated protein kinase inhibitors: where are we now and where are we going? Ann Rheum Dis 65 (Suppl III): iii83–iii88. 10.1136/ard.2006.058388 PMC1798373 17038480

[49] Piccinini AM, Midwood KS (2012) Endogenous control of immunity against infection: tenascin-C regulates TLR4-mediated inflammation via microRNA-155. Cell Rep 2: 914–926. 23084751 10.1016/j.celrep.2012.09.005 PMC3607221

[50] Miyake K (2006) Roles for accessory molecules in microbial recognition by Toll-like receptors. J Endotoxin Res 12: 195–204. 16953972 10.1179/096805106X118807

[51] Park BS, Song DH, Kim HM, Choi BS, Lee H, et al (2009) The structural basis of lipopolysaccharaide recognition by the TLR4-MD-2 complex. Nature 458: 1191–1195. 19252480 10.1038/nature07830

[52] Stewart CR, Stuart LM, Wilkinson K, van Gils JM, Deng J, et al (2010) CD36 ligands promote sterile inflammation through assembly of a Toll-like receptor 4 and 6 heterodimer. Nat Immunol 11: 155–161. 20037584 10.1038/ni.1836 PMC2809046

[53] Taylor KR, Yamasaki K, Radek KA, DiNardo A, Goodarzi H, et al (2007) Recognitioin of hyaluronan released in sterile injury involves a unique receptor complex dependent on toll-like receptor 4, CD44, and MD-2. J Biol Chem 282: 18265–18275. 17400552 10.1074/jbc.M606352200

[54] Kelsh RM, McKeown-Longo PJ (2013) Topographical changes in extracellular matrix: Activation of TLR4 signaling and solid tumor progression. Trends in Cancer Res 9: 1–13. 24634571 PMC3952558

[55] Schor SL, Schor AM (2001) Phenotypic and genetic alterations in mammary stroma: implications for tumour progression. Breast Cancer Res 3: 373–379. 11737888 10.1186/bcr325 PMC138703

[56] Pan Q, Chathery Y, Wu Y, Rathore N, Tong RK, et al (2007) Neuropilin-1 binds to VEGF_121_ and regulates endothelial cell migration and sprouting. J Biol Chem 282: 24049–24056. 17575273 10.1074/jbc.M703554200

[57] Homandberg GA (1999) Potential regulation of cartilage metabolism in osteoarthritis by fibronectin fragments. Front Biosci 4: D713–D730. 10525477 10.2741/homandberg

[58] Morla A, Ruoslahti E (1992) A fibronectin self-assembly site involved in fibronectin matrix assembly: reconstruction in a synthetic peptide. J Cell Biol 118: 421–429. 1629240 10.1083/jcb.118.2.421 PMC2290042

[59] Hocking DC, Sottile J, McKeown-Longo PJ (1994) Fibronectin's III-1 module contains a conformation-dependent binding site for the amino-terminal region of fibronectin. J Biol Chem 269: 19183–19187. 8034677

[60] Hocking DC, Kowalski K (2002) A cryptic fragment from fibronectin's III1 modulate localizes to lipid rafts and stimulates cell growth and contractility. J Cell Biol 158: 175–184. 12105189 10.1083/jcb.200112031 PMC2173025

[61] Hocking DC, Titus PA, Sumagin R, Sarelius IH (2008) Extracellular matrix fibronectin mechanically couples skeletal muscle contraction with local vasodilation. Circ Res 102: 372–379. 18032733 10.1161/CIRCRESAHA.107.158501

[62] Feaver RE, Gelfand BD, Wang C, Schwartz MA, Blackman BR (2010) Atheroprone hemodynamics regulate fibronectin deposition to create positive feedback that sustains endothelial inflammation. Circ Res 106: 1703–1711. 20378855 10.1161/CIRCRESAHA.109.216283 PMC2891748

[63] Kendal-Wright CE, Hubbard D, Gowin-Brown JH, Bryant-Greenwoord GD (2010) Stretch and inflammation-induced Pre-B cell colony-enhancing factor (PBEF/Visfatin) and interleukin-8 in amniotic epithelial cells. Placenta 31: 665–674. 20598369 10.1016/j.placenta.2010.06.007 PMC2921847

[64] Bradshaw MJ, Cheung MC, Ehrlich DJ, Smith ML (2012) Using molecular mechanics to predict bulk material properties of fibronectin fibers. PLoS Comput Biol 8 : e1002845- doi:10.1371/journal.pcbi1002845. Epub 2012 Dec 27. 10.1371/journal.pcbi.1002845 PMC3531316 23300425

[65] Deno DC, Saba TM, Lewis EP (1983) Kinetics of endogenously labeled plasma fibronectin: Incorporation into tissues. Am J Physiol 245: R564–R575. 6624952 10.1152/ajpregu.1983.245.4.R564

[66] Moretti FA, Chauhan AK, Iaconcig A, Porro F, Baralle FE, et al (2007) A major fraction of fibronectin present in the extracellular matrix of tissues is plasma-derived. J Biol Chem 282: 28057–28062. 17644525 10.1074/jbc.M611315200

[67] Oh E, Pierschbacher M, Ruoslahti E (1981) Deposition of plasma fibronectin in tissues. Proc Natl Acad Sci 78: 3218–3221. 6789333 10.1073/pnas.78.5.3218 PMC319532

[68] Thompson C, Blumenstock FA, Saba TM, Feustel PJ, Kaplan JE, et al (1989) Plasma fibronectin synthesis in normal and injured humans as determined by stable isotope incorporation. J Clin Invest 84: 1226–1235. 2794059 10.1172/JCI114289 PMC329782

[69] Booth AJ, Wood SC, Cornett AM, Dreffs AA, Lu G, et al (2012) Recipient-derived EDA fibronectin promotes cardiac allograft fibrosis. J Pathol 226: 609–618. 21960174 10.1002/path.3010 PMC3991242

[70] Volz KS, Miljan E, Khoo A, Cooke JP (2012) Development of pluriopotent stem cells for vascular therapy. Vascul Pharmacol 56: 288–296. 22387745 10.1016/j.vph.2012.02.010 PMC3595194

[71] Arslan F, Smeets MB, Riem Vis PW, Karper JC, Quax PH, et al (2011) Lack of fibronectin-EDA promotes survival and prevents adverse remodeling and heart function deterioration after myocardial infarction. Circ Res 108: 582–592. 21350212 10.1161/CIRCRESAHA.110.224428

[72] Khan MM, Gandhi C, Chauhan N, Stevens JW, Motto DG, et al (2012) Alternatively-spliced extra domain A of fibronectin promotes acute inflammation and brain injury after cerebral ischemia in mice. Stroke 43: 1376–1382. 22363055 10.1161/STROKEAHA.111.635516 PMC3335936

[73] Hirshoren N, Kohan M, Assayag M, Neuman T, Vernea F, et al (2013) Extra domain-A fibronectin is necessary for the development of nasal remodeling in chronic allergen-induced rhinitis. Ann Allergy Asthma Immunol 110: 322–327. 23622001 10.1016/j.anai.2013.03.002

[74] van der Straaten HM, Canninga-van Dijk MR, Verdonck LF, Castigliego D, Borst HP, et al (2004) Extra-domain-A fibronectin: a new marker of fibrosis in cutaneous graft-versus-host disease. J Invest Dermatol 123: 1057–1062. 15610514 10.1111/j.0022-202X.2004.23474.x

[75] Mogami H, Kishore AH, Shi H, Keller PW, Akgul Y, et al (2013) Fetal fibronectin signaling induces matrix metalloproteases and cyclooxygenase-2 (COX-2) in amnion cells and preterm birth in mice. J Biol Chem 288: 1953–1966. 23184961 10.1074/jbc.M112.424366 PMC3548503

